# Computationally guided enzyme engineering for regioselective synthesis of fucosylated human milk oligosaccharides

**DOI:** 10.1007/s00253-026-13860-8

**Published:** 2026-05-14

**Authors:** Yaya Yang, Aitor Vega, Jesper Holck, Antoni Planas, Xevi Biarnés, Birgitte Zeuner

**Affiliations:** 1 Section for Protein Chemistry and Enzyme Technology, Department of Biotechnology and Biomedicine, Technical University of Denmark, Kgs. Lyngby, Denmark; 2 Laboratory of Biochemistry, Institut Químic de Sarrià, Universitat Ramon Llull, Barcelona, Spain; 3 Chemistry Section, Royal Academy of Sciences and Arts of Barcelona, Barcelona, Spain

**Keywords:** Fucosidase, Transglycosylation, Protein engineering, Human milk oligosaccharides, Computational design

## Abstract

**Abstract:**

Human milk oligosaccharides (HMOs) are key bioactive components of human milk that support infant health and microbiome development. Prevalent HMOs include the internally fucosylated pentasaccharides lacto-*N*-fucopentaose II (LNFP II) and lacto-*N*-fucopentaose III (LNFP III), which are absent from infant formula. Their enzymatic synthesis from simpler HMOs such as 3-fucosyllactose (3FL), lacto-*N*-tetraose (LNT) and lacto-*N*-neotetraose (LNnT) represents an important step towards bridging this gap, especially now that these simpler HMOs are available on an industrial scale. We evaluated the use of the GH29B α-1,3/4-L-fucosidase *Sp*GH29^C^ from *Streptococcus pneumoniae* for transfucosylation at equimolar donor-to-acceptor ratio and applied the computational pipeline *BindScan* to design variants with reduced hydrolytic activity to avoid undesirable product hydrolysis. Guided by these predictions, we generated and tested 21 variants of *Sp*GH29^C^, achieving significantly reduced hydrolysis and enhanced transglycosylation yields. Variants W264F and D257N reached LNFP II yields of up to 73% and 68%, respectively, while A173H improved LNFP III formation to 53%. Importantly, the product levels remained stable over 24 h as the variants displayed significantly decreased product hydrolysis as intended. Further binding analyses with *BindScan* enabled rational targeting of regioselectivity, identifying W211 as a key position influencing branched vs. linear product formation for LNFP II synthesis, while F202 and D257 variants improved regioselectivity in LNFP III synthesis. This study demonstrates that computationally guided protein engineering can optimize glycosidase-catalyzed transglycosylation and provides a framework for designing regioselective biocatalysts for complex oligosaccharides synthesis.

**Key points:**

• *BindScan designs fucosidase variants with improved transglycosylation performance*

• *SpGH29*^*C*^ *variants efficiently synthesize LNFP II and LNFP III with low hydrolysis*

• *SpGH29*^*C*^* positions W211, F202 and D257 influence regioselectivity*

**Supplementary information:**

The online version contains supplementary material available at 10.1007/s00253-026-13860-8.

## Introduction

Human milk provides essential nutrients and bioactive compounds that shape microbial colonization and development during infancy (Walker 2010; Dinleyici et al. 2023) and breastfed infants show reduced risk of respiratory, diarrheal, and inflammatory diseases, underscoring the gut microbiome’s crucial role (Victora et al. 2016; Ho et al. 2018). Investigations into the components and functions of human milk have pointed towards an important role of the human milk oligosaccharides (HMOs) (Bode 2012; Faijes et al. 2019), which may also have positive effects on adults (Šuligoj et al. 2020; Carter et al. 2025). HMOs are lactose-based oligosaccharides and constitute the third most abundant component of human milk. Among them, fucosylated HMOs are the most abundant ones (Bode 2012; Soyyilmaz et al. 2021). Infant formula lacks HMOs because they are largely absent from cow’s milk. To bridge the current gap between infant formula and human milk, there is a large incentive to produce HMOs. Today, seven HMOs are available from cell factories in large quantities, namely the fucosylated or sialylated lactoses 2′-fucosyllactose (2’FL), 3-fucosyllactose (3FL), difucosyllactose (DFL), 3′-sialyllactose (3′SL), and 6′-sialyllactose (6′SL), and the two core HMO tetrasaccharides lacto-*N*-tetraose (LNT) and lacto-*N*-neotetraose (LNnT) (Schönknecht et al. 2023; Wichmann et al. 2026). However, as carbohydrate complexity increases, cell factory design becomes more challenging, and yields decrease (Sprenger et al. 2017; Barnum et al. 2024). Nevertheless, as more than 200 different HMOs have been identified in human milk and at least 20 of these are present in concentrations > 0.1 g/L in human milk (Soyyilmaz et al. 2021), the need remains to produce more complex HMOs. Among these are lacto-*N*-fucopentaose II (LNFP II) and lacto-*N*-fucopentaose III (LNFP III), whose non-reducing ends resemble the blood group antigens Lewis^A^ and Lewis^X^, respectively, as they feature a fucosylation at the internal GlcNAc moiety leading to a branched structure From an average HMO profile calculated from literature data on human milk composition, the concentrations of LNFP II and LNFP III in mature milk (15—90 days postpartum) were estimated to be 0.79 g/L and 0.26 g/L, respectively, placing these two HMOs in top 10 of the most abundant HMOs, when comparing molar ratios (Soyyilmaz et al. 2021). Thus, the ability to enzymatically synthesize these internally fucosylated HMOs from the simpler microbially produced HMOs 3FL, LNT, and LNnT provides an important step towards closing the gap between human milk and infant formula.

In our previous work, the recombinantly produced α−1,3/4-L-fucosidase *Sp*GH29^C^ from *Streptococcus pneumoniae* TIGR4 reached an unprecedented 91% yield of LNFP II when using 10 mM 3FL as donor and 100 mM LNT as acceptor at optimized conditions (Yang et al. 2024b). With such high yields and documented high regioselectivity, this enzyme is suitable for implementation at large scale in combination with cell factories to provide the 3FL and LNT substrates. However, using a tenfold excess of one substrate makes the process less feasible for large scale. Thus, in the current work, we assessed the enzyme at equimolar dosage of donor and acceptor substrates and employed the computational pipeline *BindScan* to design protein variants with decreased hydrolytic activity to improve the regioselective enzymatic formation of the internally fucosylated HMOs LNFP II and LNFP III. *BindScan* is a high-throughput structure-based mutational analysis pipeline that combines electrostatic potential and binding affinity metrics to rationally guide enzyme redesign for improved catalytic properties (Vega et al. 2025). Its applicability in engineering glycoside hydrolases for improved transglycosylation has previously been demonstrated for enzymes from GH20 and GH51 (Bissaro et al. 2015a; Vega et al. 2025); here, we expand the evidence into GH29.

## Materials and methods

### Modeling the transglycosylation intermediates of *Sp*GH29^C^

The structure of the transglycosylation intermediates of *Sp*GH29^C^ was modelled as in (Bissaro et al. 2015a; Vega et al. 2025). The covalent fucosyl-nucleophile intermediate of *Sp*GH29^C^ was modelled from the crystallographic structures of this enzyme in complex with Lewis^A^ (PDB accession code: 6OR4) and the fucosyl-nucleophile intermediate of the close ortholog *Bacteroides thetaiotaomicron* GH29 (PDB accession code: 2WVS). The Lewis^A^ substrate was removed from 6OR4 and the Glu215Gln substitution reverted to Glu215. The nucleophile Asp171Asn sidechain from 6OR4 was substituted by the equivalent Asp229 in 2WVS covalently attached to fucose after structural superposition on the backbones of these residues. No steric clashes were detected as both initial structures already contained a fucose ring at −1 subsite. Acceptor compounds lacto-*N*-biose (Gal-β1,3-GlcNAc; LNB) and *N*-acetyllactosamine (Gal-β1,4-GlcNAc; LacNAc) were docked to this fucosyl-enzyme intermediate of *Sp*GH29^C^ with AutoDock Vina (Trott and Olson 2010). A search grid-box sized 20 × 20 × 20 Å^3^ was centered at the C1 atom of fucose, exhaustiveness level set to 24, and 20 binding modes were requested. Among the lowest energy binding modes, two different orientations of each acceptor were considered: one orientation in which the reducing end GlcNAc ring occupies the + 1 subsite, prone to fucose transfer, and a second orientation in which the non-reducing end Gal ring occupies the + 1 subsite.

### BindScan simulation set-up for *Sp*GH29^C^ variant design

The flexible ligand *BindScan* protocol described in (Vega et al. 2025) was used. Four independent simulations were run, one for each acceptor (LNB or LacNAc) and orientation (GlcNAc or Gal at subsite + 1). All amino acid positions within 15 Å of any atom of the acceptor compounds were cast, resulting in 135 positions. A full saturation mutagenesis library was built for each position separately. 30 structure replicates were modeled for each mutant using MODELLER (Šali and Blundell 1993) satisfying the spatial restraints of the acceptor in the template structure of the corresponding transglycosylation intermediate. For each modeled structure, the protein electrostatic potential gradient was measured at the coordinates of the substrate atoms involved in the glycosidic bond formation/cleavage using APBS (Jurrus et al. 2018). The positive group was defined by atoms O5, C1, and C2 of fucose, and the negative group was defined i) at the atoms O4, C4, C5 and C3 of either GlcNAc or Gal of LNB (depending on the orientation) or (ii) at the atoms O3, C3, C4 and C2 of either GlcNAc or Gal of LacNAc (depending on the orientation). The gradients were computed and averaged for all structure replicates of each mutant. For binding affinity measurements, the corresponding acceptor compound was docked again onto each modeled structure with AutoDock Vina using the same docking configuration as above. Twenty different binding modes were requested for each acceptor and the corresponding VINA binding affinity scores were stored together with their all-atom root-mean-squared deviations (RMSD) with respect to the reference orientation in the starting template structure. The binding affinities of the docked poses with minimum RMSD were averaged for all structure replicates of each mutant. Reactivity metrics (based on electrostatic potential gradients) and affinity metrics (based on lowest RMSD affinities) were represented using sensitivity bar plots. Simulations were run in the ABACO HPC cluster (IQS Barcelona) using 128 CPU cores.

### Chemicals

The HMO substrates 3-fucosyllactose (3FL), lacto-*N*-tetraose (LNT) and lacto-*N*-neotetraose (LNnT) were generously provided by DSM-Firmenich (Hørsholm, Denmark). The analytical standards of lacto-*N*-fucopentaose I (LNFP I), II (LNFP II) and III (LNFP III) were purchased from Biosynth (Compton, UK), while that of lacto-*N*-fucopentaose V (LNFP V) was from Elicityl (Crolles, France). All other chemicals were purchased from Merck (Darmstadt, Germany).

### Site-directed mutagenesis, expression and purification of fucosidase variants

The designed variants of *Sp*GH29^C^—and two variants (E215A and E215Q) targeting inactivation of the catalytic acid/base residue—were prepared via site-directed mutagenesis exactly as described previously (Yang et al. 2024b) using designed primers (Table S1). Expression of all variants was performed in *E. coli* BL21(DE3) according to our previously established protocol (Yang et al. 2024b).

### Transglycosylation reactions

Transglycosylation reactions catalyzed by the designed variants of *Sp*GH29^C^ were performed in an 80 mM phosphate buffer (pH 6.0), i.e. optimal conditions for transglycosylation as described in our previous work (Yang et al. 2024b). The reaction temperature (40 °C) and enzyme concentration (0.5 μM) were kept constant, and the starting concentrations of the donor substrate 3FL and the acceptor substrate LNT were both 100 mM. Reactions were monitored for 24 h. The WT and selected mutants (A173H, D257N, W264F, F202D and W39A) were also assessed in a similar reaction using 100 mM LNnT as acceptor substrate. To terminate the reactions, 20 μL of the reaction sample was transferred to 380 μL of preheated water to dilute it 20-fold at each time point (1, 10, 30, 60, 180, 300 and 1440 min), followed by a 10-min heating at 95 °C.

### HPAEC-PAD analysis

The products of the transglycosylation reactions were quantified using high-performance anion exchange chromatography with pulsed amperometric detection (HPAEC-PAD) on an ICS6000 system Thermo Fisher Scientific, Waltham, Massachusetts, USA). An analytical CarboPac™ PA1 column (2 mm × 250 mm) paired with a CarboPac™ PA1 guard column (2 mm × 50 mm) was employed. The analysis was conducted using an eluent system consisting of MilliQ water (A), 500 mM NaOH (B), and 500 mM NaOAc containing 0.02% (*w/v*) NaN_3_ (C) at a flow rate of 0.25 mL/min and a temperature of 30 °C. External standards of fucose, 3FL, LNFP II and lactose at concentrations of 0.001, 0.002, 0.004, 0.008, 0.01, 0.02, 0.04, and 0.05 mM were employed to generate calibration curves of hydrolysis and transglycosylation products for the LNT transfucosylation reactions, whereas mixtures of fucose, LNnT and lactose at concentrations of 0.005, 0.01, 0.05, 0.1, 0.2, 0.4 and 0.5 mM, were prepared to generate calibration curves for quantifying reaction products for the LNnT transfucosylation. The reaction products obtained from the LNT transfucosylation were separated and quantified using isocratic elution at a ratio of 88:12:0 (A:B:C, %) for 14 min. The column was then washed with a mixture of 3:12:85 (A:B:C, %) for 4 min, followed by re-equilibration at 88:12:0 (A:B:C, %) for 10 min. The products from the LNnT transfucosylation were separated and quantified using isocratic elution at a ratio of 92:8:0 (A:B:C, %) for 35 min. The column was then washed with a mixture of 7:8:85 (A:B:C, %) for 5 min, followed by re-equilibration at 92:8:0 (A:B:C, %) for 5 min. Since we could not separate the LNFP III product from 3FL by HPAEC-PAD, we quantified the LNFP III transglycosylation product based on the reduction in LNnT concentration (as compared to the negative control at all reaction times) and verified the LNFP III identity by LC–ESI–MS (below).

As previously (Yang et al. 2024b), the molar transglycosylation yield was determined as the percentage of LNFP II/III produced in the reaction compared to the initial 3FL donor concentration ([LNFP II or III]/[3FL]_0_). Initial rates of hydrolysis and transglycosylation were defined as the concentration of fucose and LNFP II/III, respectively, formed per min of reaction over the time where the product formation curve was linear.

### LC–ESI–MS analysis

Identification and quantitation of transglycosylation product regioisomers was performed using liquid chromatography coupled to electrospray ionization-mass spectrometry (LC–ESI–MS) exactly as described previously (Yang et al. 2024b), using samples from the reaction conditions and time that resulted in the highest product yield for both WT and variants. Analysis was performed in Compass TASQ2.2 (Bruker Daltonics, Bremen, Germany) using LNFP I, LNFP II and LNFP V as external standards for reactions with LNT as acceptor substrate, and LNFP III as external standard for those with LNnT. Additionally, two samples from a previous study (Yang et al. 2024a) were included as references to compare linear non-HMO LNFP regioisomers to those previously observed in transglycosylation catalyzed by fucosidases of the GH29A subfamily. The diagnostic fragments for the various LNFP structures were verified by MS^2^ according to Pfenninger et al*.* (Pfenninger et al. 2002).

### Statistics and graphics

Statistical analysis for significance (*p* < 0.05) was assessed using a one-way ANOVA and Tukey’s HSD test, both conducted with JMP® version 17.0.0 (SAS Institute Inc., Cary, NC, USA). Graphical representations of the data were created with Origin® version 9.8.0.200 (OriginLab, USA).

## Results

### Transglycosylation catalyzed by WT *Sp*GH29^C^

**Fig. 1:**
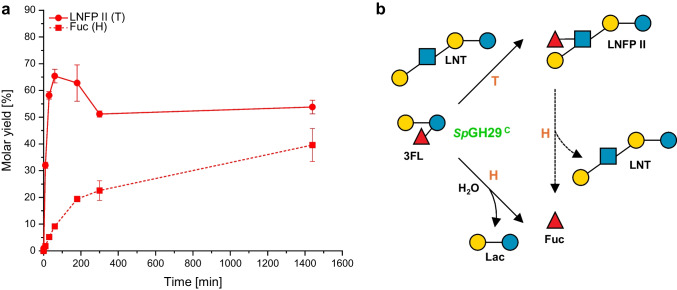
**a** Formation of the transglycosylation product lacto-*N*-fucopentaose (LNFP II) and the hydrolysis product fucose (Fuc) catalyzed by WT *Sp*GH29^C^ in the reaction on 100 mM 3-fucosyllactose (3FL) and 100 mM lacto-*N*-tetraose (LNT) given as molar yields based on the starting donor substrate concentration. **b** Scheme of the reaction displaying the desired transglycosylation (T) reaction alongside the unwanted substrate (primary) and product (secondary) hydrolysis (H) reactions. Reactions are drawn for the LNT acceptor; equivalent reactions for the lacto-*N*-neotetraose (LNnT) acceptor result in formation of LNFP III

The GH29B α−1,3/4-L-fucosidase *Sp*GH29^C^ readily catalyzes transglycosylation to form the internally fucosylated HMO lacto-*N*-fucopentaose II (LNFP II) when using the simpler HMOs 3-fucosyllactose (3FL) and lacto-*N*-tetraose (LNT) as donor and acceptor substrate, respectively (Yang et al. 2024b) (Fig. 1b). Here, we tested the transglycosylation efficiency and changes in regioselectivity at an acceptor-to-donor (A:D) ratio of 1, using 100 mM of each substrate at the previously optimized reaction conditions (Yang et al. 2024b), namely pH 6.0 in 80 mM phosphate buffer. Under these conditions, WT *Sp*GH29^C^ reached a transient maximum lacto-*N*-fucopentaose II (LNFP II) yield of 65% after 1 h of reaction (Fig. 1a). At this reaction time point, the release of fucose is 9%, indicating that competing substrate hydrolysis takes place (Fig. 1). After 5 h of reaction the total LNFP II yield decreases to a steady level around 50%, suggesting an equilibrium between donor conversion, LNFP II formation and secondary hydrolysis of the product. Even at higher donor conversions (fucose release up to 40% after 24 h) LNFP II formation does not increase.

### Computational variant design

**Fig. 2:**
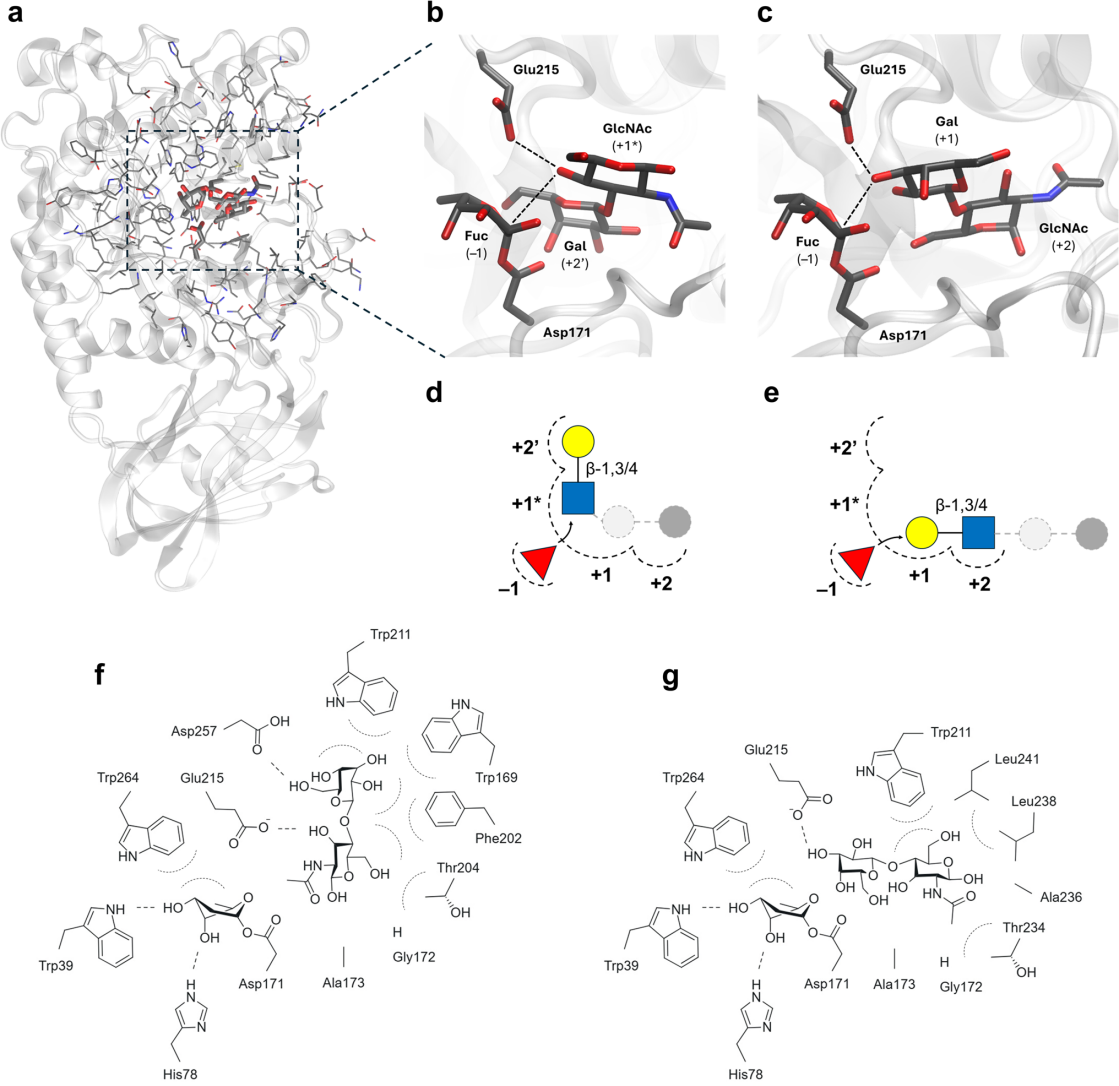
Three-dimensional structure model and *BindScan* simulations of the fucosyl-nucleophile covalent intermediate of *Sp*GH29^C^ in complex with a lacto-*N*-biose (LNB) and *N*-acetyllactosamine (LacNAc) in the orientation to render internally fucosylated, i.e. branched, LNFP II (**b**, **d**) and a linear LNFP III isomer (**c**, **e**), respectively. **a** Full protein view. Positions mutated during the *BindScan* simulation are shown in thick lines (all residues within 15 Å of the acceptor). **b** Magnified representation of the catalytic site for LNB and **c** for LacNAc. Catalytic residues are labeled as Asp171 (nucleophile) and Glu215 (acid/base). The protein backbone is represented as cartoon in transparent gray. **d**, **e** Schematic representations of the two different binding modes of the acceptors LNB or LacNAc used for the four *BindScan* simulations to render either internally fucosylated (branched; **d**) or terminally fucosylated (linear; **e**) isomers. The reducing end lactose moiety of LNT and LNnT acceptors, used experimentally, was not included in the calculations and is shown here (in gray) only for visualization purposes of the potential product isomers. Subsite nomenclature as suggested for *Sp*GH29^C^ (Hobbs et al. 2019). **f**, **g** Enzyme–substrate interactions at the active site of the modelled covalent fucosyl-enzyme intermediate in complex with LacNAc in the two explored binding modes: **f** orientation leading to the branched isomer (similar to **d**) and **g** to a linear isomer (similar to **e**). Straight dashed lines indicate hydrogen bonds, while curved dashed lines indicate hydrophobic interactions

A high-throughput screening campaign on an extended library of site saturation mutants of *Sp*GH29^C^ was performed in silico with *BindScan* aimed at identifying sensible spots to modulate hydrolytic activity and transglycosylation efficiency for production of the internally fucosylated HMOs LNFP II and LNFP III. The simulations took advantage of the crystallographic structures of *Sp*GH29^C^ in complex with Lewis^A^, which resembles the non-reducing end trisaccharide of the LNFP II product, and with Lewis^X^, which resembles the non-reducing end trisaccharide of LNFP III and is similar to the 3FL donor substrate (Lewis^X^ is similar to 3FL with an N-acetylglucosamine, GlcNAc, core instead of glucose) (Hobbs et al. 2019). Based on this, we prepared a model of the fucosyl-nucleophile covalent intermediate in complex with acceptor substrates: lacto-*N*-biose (Gal-β1,3-GlcNAc; LNB) and *N*-acetyllactosamine (Gal-β1,4-GlcNAc; LacNAc) (Fig. 2b–c). These disaccharide units correspond to the non-reducing ends of the larger acceptor substrates lacto-*N*-tetraose (LNT) and lacto-*N*-neotetraose (LNnT), respectively. The predicted structures of the complexes are compatible with the transglycosylation reaction, placing the O4/O3 oxygen of the acceptor residue (GlcNAc) close to both the general acid/base residue (E215) and the anomeric carbon of the donor fucose (Fuc) (Fig. 2).

**Fig. 3:**
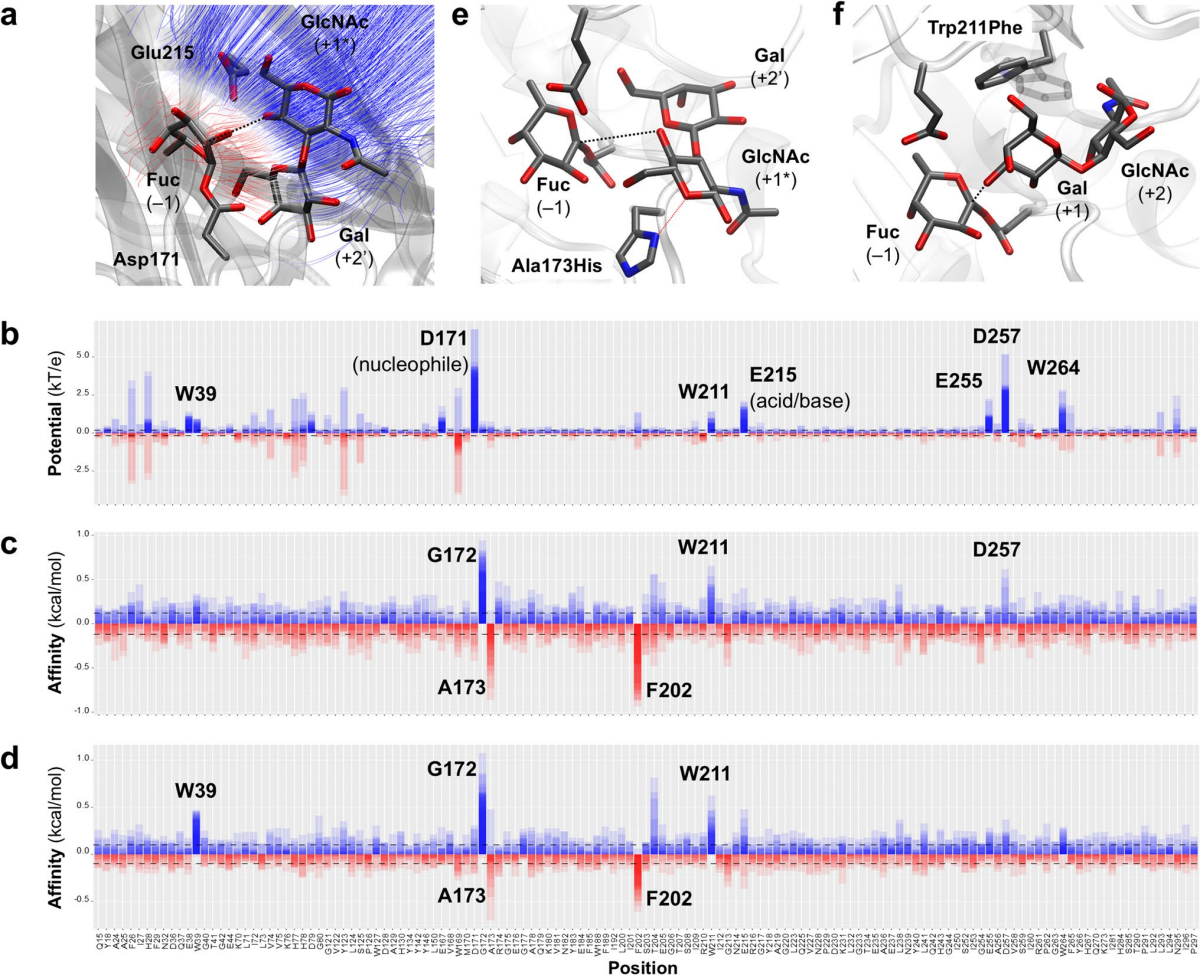
**a** Protein electrostatic field lines at the active site of *Sp*GH29^C^ colored by electrostatic potential value, from − 28 (red) to − 20 (blue) kT/e. The acceptor substrate LNB is shown for reference, but it was not present during the electrostatic potential calculation. **b** *BindScan* prediction of mutation spots on *Sp*GH29^C^ that modulate the electrostatic potential gradient at the active site, **c** binding of the LNB acceptor in the orientation leading to the LNFP II isomer, and **d** binding of LacNAc acceptor in the orientation leading to the LNFP III isomer. For each position, deviations from the reference metric of the wild type are shown in bar plots (gain of function in red, loss of function in blue). Dotted lines in the bar plot represent the thresholds for significant deviations (± 2.5 times standard deviation of wild-type metric). **e** Magnified representation of the catalytic site for the A173H mutant in complex with LNB in the LNFP II isomer formation orientation and **f** for the W211F mutant in complex with LacNAc in the linear isomer formation orientation

We first evaluated the electrostatic potential exerted by the protein at the catalytic center of *Sp*GH29^C^. As we have recently shown to be common to many glycoside hydrolases (Vega et al. 2025), there is a clear electrostatic gradient exerted by the protein at the active site (Fig. 3a) that fosters substrate charge separation along the glycosidic bond to be cleaved. The field lines run parallel to the location of the scissile glycosidic bond, and the electrostatic potential values are complementary to the substrate charge separation developed at the transition state (TS) of the hydrolytic reaction, thus contributing to its stabilization. Thus, we used the recently introduced electrostatic potential gradient score (Vega et al. 2025) to identify protein positions that modulate the hydrolytic activity of *Sp*GH29^C^ by altering the protein electrostatic properties at the active site. The gradient score evaluates the difference in electrostatic potential exerted by the protein at both sides of the scissile glycosidic bond. When the gradient is reduced, a decrease in hydrolytic activity of the enzyme can be expected because of a lesser electrostatic complementarity to the TS. The *BindScan* simulation revealed a list of protein spots that notably decrease the electrostatic potential gradient (blue bars in Fig. 3d) indicating electrostatic destabilization of the TS of the hydrolytic reaction. The heatmap of the full in silico mutation dataset is provided in Fig. S1. In GH29, the position of the catalytic nucleophile residue is easily determined from conservation in a multiple sequence alignment, while the acid/base assignment is only straightforward for enzymes belonging to subfamily B like *Sp*GH29^C^ (Shaikh et al. 2013; Zeuner et al. 2020). The catalytic nucleophile (D171) and acid/base (E215) residues both appear as clear cold spots reducing the electrostatic potential gradient, as consistently observed in this kind of simulations. Accordingly, mutants at these essential positions render protein variants in which the hydrolytic activity is hampered or even abolished; indeed, our mutants E215A and E215Q (Fig. S2) were completely devoid of both hydrolytic and transglycosylation activity. Interesting spots predicted to reduce hydrolytic activity are W39, W211, E255, D257 and W264. Thus, we designed the mutants at these positions to evaluate their effect on transglycosylation efficiency: W39A, W39Q, W39Y, W211A, W211H, W211F, W211R, E255K, E255Q, D257N, D257R, W264F, W264H, W264A and W264R.

Further, we wanted to investigate whether we could also improve transglycosylation efficiency by selecting variants predicted by *BindScan* to alter acceptor binding, as has been observed for other engineered glycoside hydrolases (Bissaro et al. 2015a; Castejón-Vilatersana et al. 2021). At the same time, we wondered if it was possible to modify regioselectivity of the transglycosylation reaction in *Sp*GH29^C^. To this end, we performed four *BindScan* simulations to identify positions that modulate the binding of the LNB and LacNAc acceptor substrates in different orientations to render different regioisomers of LNFP II and LNFP III. Two simulations were performed with the GlcNAc ring located at the + 1*subsite (Fig. 2d), yielding the desired LNFP II and LNFP III, and two simulations with the Gal ring placed at the + 1 subsite (Fig. 2e) yielding the corresponding linear regioisomers. Heatmaps of both full in silico mutation datasets are provided in Fig. S1.

The simulations identified positions A173 and F202 to improve binding of both acceptor substrates in the orientation leading to the formation of the true LNFP II and LNFP III isomers (hotspots as red bars in Fig. 3c-–d and Table 1). Mutations at these positions may lead to an overall increase of transglycosylation efficiency via improved acceptor binding. To test this hypothesis, we designed the variants A173H, A173D, F202D, and F202S. Interestingly, *BindScan* identified G172, W211 and D257 as positions that upon mutation disfavor the formation of the LNFP II structure (cold spots as blue bars in Fig. 3c), potentially leading to the formation of other regioisomers (Table 1). Thus, we expanded the selected designs to also include the following variants: G172L and G172W. W211A, W211H, W211F and W211R were already designed based on the effect electrostatic potential gradient (see above). Other positions identified by *BindScan* to favor the binding of the acceptor substrate in alternative orientations were G213 and L241 (Table 1), but these had no predicted effect on the formation of true LNFP II nor LNFP III and were not considered further for investigation.

**Table 1:** Sequence positions of *Sp*GH29^C^ identified by *BindScan* to either improve (+) or disfavor (-) the binding of LNB and LacNAc acceptors or display no effect in binding (o). Two different acceptor orientations were considered: fucose transfer to the internal GlcNAc (for formation of the desired, branched LNFP II and LNFP III; Fig. 2d) or to the non-reducing end Gal (for linear isomers; Fig. 2e)

Acceptor	LNB		LacNAc	
Fucose accepting unit	GlcNAc	Gal	GlcNAc	Gal
Expected product	LNFP II	linear isomer	LNFP III	linear isomer
W39*	o	-	-	-
G172	-	+	-	-
A173	+	+	+	+
F202	+	o	+	o
W211*	-	-	-	+
G213	o	+	o	o
L241	o	o	o	+
E255*	o	o	o	o
D257*	-	o	o	+
W264*	o	o	-	o

Sequence positions predicted to destabilize the hydrolytic reaction are marked with *

### Hydrolytic activities of *Sp*GH29^C^ variants and synthesis of LNFP II with LNT as acceptor

The WT *Sp*GH29^C^ and all variants were recombinantly expressed in *E. coli* BL21(DE3) in yields of 15–118 mg/L (Table S2) and purified via their His_6_-tag to high purity as assessed by SDS-PAGE (Fig. S2).

**Fig. 4:**
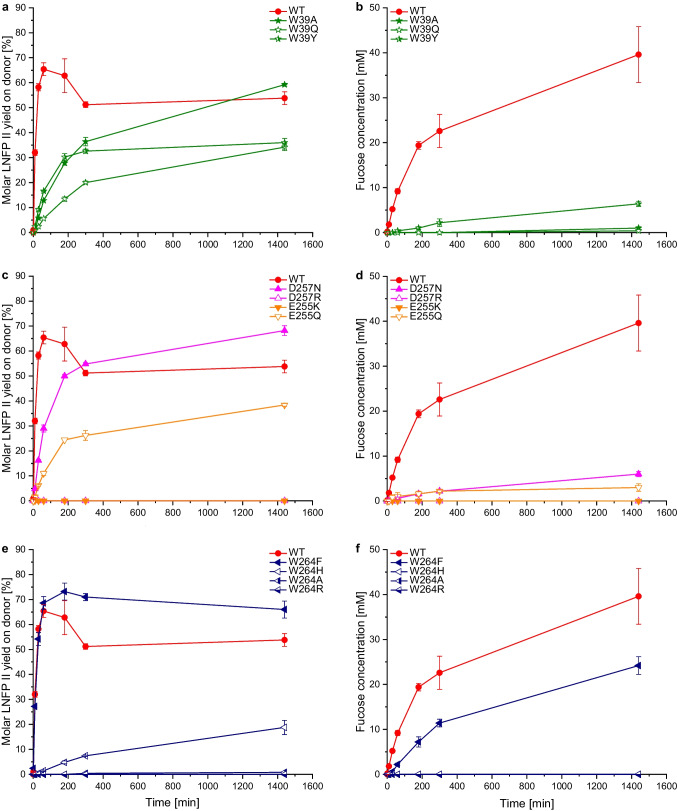
Screening of transfucosylation and hydrolysis catalyzed by *Sp*GH29^C^ WT (red) and four of its variants designed based on electrostatic potential targeting W39 (green; **a**–**b**), E255 (orange) and D257 (pink; **c**–**d**), and W264 (navy; **e**–**f**). Transfucosylation (**a**, **c**, **e**) is indicated as molar transglycosylation yield ([LNFP II]/[3FL]_0_), whereas hydrolysis (**b**, **d**, **f**) is shown as released fucose from the reactions using 100 mM 3FL and 100 mM LNT (*n* = 2)

**Fig. 5:**
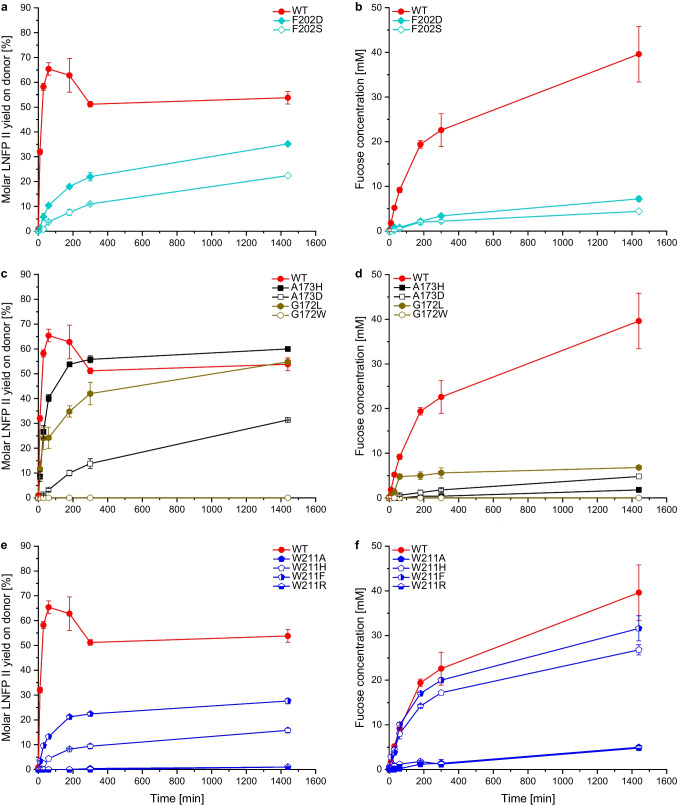
Screening of transfucosylation and hydrolysis catalyzed by *Sp*GH29^C^ WT (red) and its variants designed based on binding affinity targeting F202 (cyan; **a**–**b**), A173 (black) and G172 (brown; **c**–**d**), and finally W211 (blue; **e**–**f**) designed based on electrostatics. Transfucosylation (**a**, **c**, **e**) is indicated as molar transglycosylation yield ([LNFP II]/[3FL]_0_), whereas hydrolysis (**b**, **d**, **f**) is shown as released fucose from the reactions using 100 mM 3FL and 100 mM LNT (*n* = 2)

The hydrolytic behavior and transglycosylation efficiency of the *Sp*GH29^C^ variants differs from those of the WT (Fig. 4; Fig. 5; Table 2). In many variants, the release of fucose resulting from hydrolysis was limited (below 5% at 24 h for variants at positions W39, A173, G172, F202, D257 and E255). In fact, hydrolysis rates were significantly lowered for almost all variants (Table 2). Despite the low hydrolysis rates, the LNFP II formation yields were notably high for some of these mutants: 55% for G172L, 59% for W39A, 60% for A173H and 68% for D257N (Table 2).

**Table 2:** Initial transfucosylation rate (*r*_T_), initial hydrolysis rate (*r*_H_), the ratio of initial transfucosylation rate to initial hydrolysis rate (T/H), and maximum LNFP II yield ([% molar yield on donor]) at corresponding time points [h] for WT *Sp*GH29^C^ and its variants. Reactions utilized 100 mM 3FL as donor substrate and 100 mM LNT as acceptor substrate (*n* = 2). For variants marked with an asterisk, LC–ESI–MS analysis (Fig. S3) detected LNFP regioisomer levels > 5% at the time of the maximum yield (percentage of regioisomers out of total LNFP yield (Table S3) is given in parentheses)

*Sp*GH29^C^ variant	*r*_T_ [μM∙min^−1^]	*r*_H_ [μM∙min^−1^]	T/H	Max. yield of LNFP II* [%]	Time [h]
WT	1950 ± 52^a^	107 ± 4.9^c^	18 ± 1^cd^	65 ± 3^b^	1
W39A	156 ± 4.9^efg^	0.70 ± 0.20^e^	230 ± 58^b^	59 ± 0.6^c^	24
W39Q	67.6 ± 2.6^ghij^	0.28 ± 0^e^	240 ± 9^b^	34 ± 1^de^	24
W39Y	168 ± 8.5^ef^	4.47 ± 0.43^e^	38 ± 6^cd^	36 ± 2^de^	24
G172L	125 ± 7.1^fghi^	18.5 ± 0.14^e^	6.8 ± 0.4^cd^	55 ± 0^c^	24
G172W	ND	ND	-	ND	-
A173D	48.1 ± 4.1^hij^	3.25 ± 0.075^e^	15 ± 2^ cd^	31 ± 0.3^ef^	24
A173H	689 ± 24^c^	1.59 ± 0^e^	450 ± 15^a^	60 ± 0.6^c^	24
F202D	178 ± 6.5^ef^	11.3 ± 1.1^e^	16 ± 1^cd^	35 ± 0.6^de^	24
F202S	37.2 ± 0.73^ij^	8.55 ± 0.46^e^	4.4 ± 0.1^cd^	22 ± 0^gh^	24
W211A	0.710 ± 0.20^j^	3.48 ± 0.17^e^	0.21 ± 0.1^d^	1.0 ± 0.3^j^* (15%)	24
W211F	228 ± 1.1^e^	156 ± 12^a^	1.5 ± 0.1^d^	28 ± 1^ fg^* (6.1%)	24
W211H	73.6 ± 1.1^ghij^	132 ± 20^b^	0.56 ± 0.1^d^	16 ± 0.8^i^* (5.2%)	24
W211R	0.700 ± 0.20^j^	3.03 ± 0.45^e^	0.23 ± 0.03^d^	1.0 ± 0.3^j^* (13%)	24
E255K	ND	ND	-	ND	-
E255Q	136 ± 0.61^fgh^	7.20 ± 0.55^e^	19 ± 1^cd^	38 ± 0.6^d^	24
D257N	494 ± 21^d^	7.72 ± 0.67^e^	64 ± 8^c^	68 ± 2^ab^	24
D257R	ND	ND	-	ND	-
W264A	0.57 ± 0^j^	ND	-	0.80 ± 0^j^	24
W264F	1790 ± 87^b^	39.2 ± 0.67^d^	46 ± 1^cd^	73 ± 3^a^* (5.2%)	3
W264H	25.6 ± 1.4^j^	ND	-	19 ± 3^hi^	24
W264R	ND	ND	-	ND	-

*ND* not detected (below detection limit). ^a–j^Differences in superscript letters indicate significant difference (*p* < 0.05) between the variants for each parameter

In contrast, variants at position W211 maintained high levels of hydrolytic activity (Table 2), despite the *BindScan* prediction (subtle cold spot in Fig. 3b). Consequently, the transglycosylation yield was reduced to 16–28% for W211H and W211F, probably because secondary hydrolysis was still significant. Interestingly, W264F also only slightly reduced the hydrolytic profile as compared to WT, but this mutant achieved a maximum 73% LNFP II yield at 3 h, which remained stable around 70% between 5 and 24 h (Fig. 4e-–f), making it the best *Sp*GH29 variant.

Several of the tested variants were (practically) inactive, and these were variants with quite drastic amino acid substitutions, namely G172W, W211A, W211R, E255K, D257R, W264A and W264R (Table 2).

### Synthesis of LNFP II regioisomers

The total LNFP II product formation was quantified by HPAEC-PAD. However, this method did not separate LNFP II from potential regioisomers; thus, we employed separation on a porous graphitized carbon (PGC) column on LC–ESI–MS to assess the presence of regioisomers. The yields reported in Fig. 4, Fig. 5, and Table 2 are the sum of regioisomers as determined by HPAEC-PAD, with the percentage of non-LNFP II regioisomers as estimated by LC–ESI–MS indicated within brackets for the few variants where non-negligible levels of regioisomers were observed (Table 2). As previously reported (Yang et al. 2024b), WT *Sp*GH29^C^ was highly regioselective, and the same was true for most of the variants as the sum of other isomers detected by LC–ESI–MS represented < 4% of the total yield (Table S3). Interestingly, for variants targeting W211, we observed significant formation of different regioisomers (Table 2; Table S3). The product isomer obtained with W211 variants (named iso3) was fucosylated at the non-reducing end Gal and was also previously observed in GH29A-catalyzed transfucosylation (Yang et al. 2024a). The formation of this linear isomer (iso3) represented 14% of the maximum yield for W211A and 12% for W211R (Fig. S3; Table S3), whereas it was 4–5% for W211H and W211F. This linear isomer (iso3) also represented 3.3% of the maximum yield for W264F, where we also observed a 1.5% yield of an isomer (iso6; Fig. S3; Table S3), which judging from its shorter retention time may well be a branched, i.e. internally fucosylated, isomer. Formation of iso6 was also observed for G172L, though not in significant amounts (Fig. S3; Table S3). Although other branched isomers were not directly targeted in the *BindScan* simulations, the predicted decrease in acceptor binding for mutations at both G172 and W264 (cold spots in Fig. 3c–d) may instead have manifested itself in the observed lower T/H as compared to WT (Table 2).

### Synthesis of LNFP III with LNnT as acceptor

**Fig. 6:**
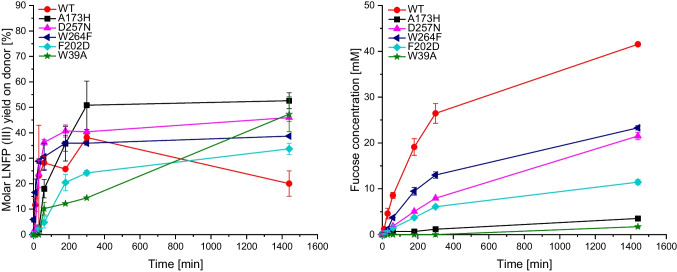
Molar yields of LNFP III and regioisomers resulting from transglycosylation (left) and fucose released from hydrolysis (right) catalyzed by *Sp*GH29^C^ WT (red circle) and selected variants W39A (green star), A173H (black square), F202D (cyan diamond), D257N (pink triangle), and W264F (navy triangle) over 24 h of reaction using 100 mM 3FL and 100 mM LNnT (*n* = 2)

Using LNnT, which was among the first two HMOs to be available in large scale from cell factories (Bych et al. 2018), as acceptor substrate, presents the opportunity to form lacto-*N*-fucopentaose III (LNFP III) as transglycosylation product (Zeuner et al. 2018). *BindScan* simulations using LacNAc as acceptor substrate (surrogate of LNnT) revealed the same hot spots for acceptor binding as for LNB (surrogate of LNT), namely A173 and F202, as well as the cold spots G172 and W211 that remained the same, but W39 appeared as a new cold spot, while D257 did not (Fig. 3d, Table 1). Thus, we assessed the performance of the *Sp*GH29^C^ WT and selected variants W39A, A173H, F202D, D257N and W264F, which all readily synthesized LNFP II via transglycosylation in the reaction with LNT (Fig. 6).

The LNFP III product formation was quantified as reduction in LNnT concentration by HPAEC-PAD (as we could not separate LNFP III from 3FL), which did thus not provide information on regioisomer formation. Again, we used separation on the PGC column on LC–ESI–MS to assess the presence of regioisomers (Fig. S4), resulting in the detection of small amounts of isomers iso8 and iso9, which both according to retention time and MS^2^ fragmentation appeared to be linear, terminally fucosylated regioisomers, i.e. Fuc-1,x-LNnT (Fig. S5). Based on peak areas, LNFP regioisomer levels > 5% were determined for WT, W39A, A173H and W264F (Table 3; Table S4).

**Table 3:** Initial transfucosylation rate (*r*_T_), initial hydrolysis rate (*r*_H_), the ratio of initial transfucosylation rate to initial hydrolysis rate (T/H), and maximum LNFP III yield ([% molar yield on donor]) at corresponding time points [h] for WT *Sp*GH29^C^ and selected variants. Reactions utilized 100 mM 3FL as donor substrate and 100 mM LNnT as acceptor substrate (*n* = 2). For variants marked with an asterisk, LC–ESI–MS analysis (Fig. S4) detected LNFP regioisomer levels > 5% at the time of the maximum yield (percentage of regioisomers out of total LNFP yield (Table S4) is given in parentheses)

*Sp*GH29^C^ variant	*r*_T_ [μM∙min^−1^]	*r*_H_ [μM∙min^−1^]	T/H	Max. yield of LNFP III* [%]	Time [h]
WT	480 ± 126^bc^	89.6 ± 9.2^a^	5.3 ± 1^c^	38 ± 1^bc^* (7.8%)	5
W39A	32.0 ± 4.5^e^	1.21 ± 0.077^e^	26 ± 2^b^	47 ± 7^ab^* (6.5%)	24
A173H	287 ± 63^cd^	3.65 ± 0.51^de^	78 ± 6^a^	53 ± 3^a^* (13%)	24
F202D	116 ± 19^de^	20.3 ± 0.30^cd^	5.7 ± 1^c^	34 ± 2^c^	24
D257N	645 ± 1.8^b^	27.0 ± 0.64^c^	24 ± 1^b^	46 ± 2^abc^	24
W264F	879 ± 18^a^	53.5 ± 4.9^b^	16 ± 1^bc^	39 ± 0^bc^* (5.4%)	24

^a–j^Difference in superscript letters indicate significant difference (*p* < 0.05) between the variants for each property

The WT reached a transient transglycosylation yield of 38% after 5 h of reaction, which was reduced to 20% after 24 h of reaction (Fig. 6). The rate and degree of hydrolysis was similar to that observed in the formation of LNFP II, but the rate of transglycosylation was approx. 4 times lower (Table 3; Table 2), ultimately resulting in lower product yields and a higher degree of product hydrolysis.

In terms of yield and T/H, A173H was the best performing variant with a transglycosylation product yield of 51% after 5 h of reaction, which slightly increased to 53% after 24 h of reaction (Fig. 6; Table 3). W39A and D257N had similar maximum yields and T/H but arrived at this maximum of 46–47% at different speeds as W39A had much lower reaction rates (Fig. 6; Table 3). D257N was the most regioselective of the variants in this reaction (Table S4), giving it an advantage in the regioselective production of LNFP III. D257N and W264F both displayed higher *r*_T_ values than the WT, and both also displayed moderate hydrolysis rates. Interestingly, D257N seemed much more prone to hydrolyze the LNFP III product than the LNFP II product (Fig. 6; Fig. 4c–d; Table 3; Table 2). F202D showed no improvement in transglycosylation over the WT, but was more regioselective and did not exhibit a transient product maximum within the monitored reaction time (Fig. 6).

## Discussion

The wild-type *Sp*GH29^C^ fucosidase has a significant transglycosylation activity (65% maximum LNFP yield) and shows significant secondary hydrolysis, with the product being hydrolyzed just after 1-h reaction. Modulation of hydrolytic activity is important to achieve high transglycosylation yields (Bissaro et al. 2015b; Zeuner et al. 2018; Castejón-Vilatersana et al. 2021), and transient product maxima as observed here requires strict control of reaction time to avoid product loss through secondary hydrolysis (Fig. 1a), which is unfavorable in large-scale manufacturing. Thus, we first aimed to reduce the hydrolytic activity of *Sp*GH29^C^ by protein engineering. We designed enzyme mutants guided by the predictions with the *BindScan* simulation protocol (Vega et al. 2025) for transglycosylation design. The rational computationally assisted engineering strategy developed here is to reduce the hydrolase activity (the *BindScan* electrostatic potential score) and increase acceptor binding (the *BindScan* binding score). The electrostatic potential guided variants may exhibit a reduction not only in secondary hydrolysis, but also in primary hydrolysis; in this case, the combined *BindScan* approach has the advantage that efficient transglycosylation may be recovered by increasing the affinity of the acceptor in the second step of the reaction via the designs guided by the binding affinity metric.

We used a model of the fucosyl-nucleophile covalent intermediate in complex with two different disaccharide acceptor substrates, despite using tetrasaccharide acceptor substrates in the transglycosylation reaction. In the crystallization of a related GH29B α−1,3/4-L-fucosidase, *Bi*AfcB, it was found the reducing-end lactose part of LNFP II did not interact significantly with the protein structure (Sakurama et al. 2012), suggesting that it is indeed sufficient to model LNB and LacNAc disaccharides in these simulations as surrogates of the larger acceptor compounds used in the experimental assays.

**Fig. 7:**
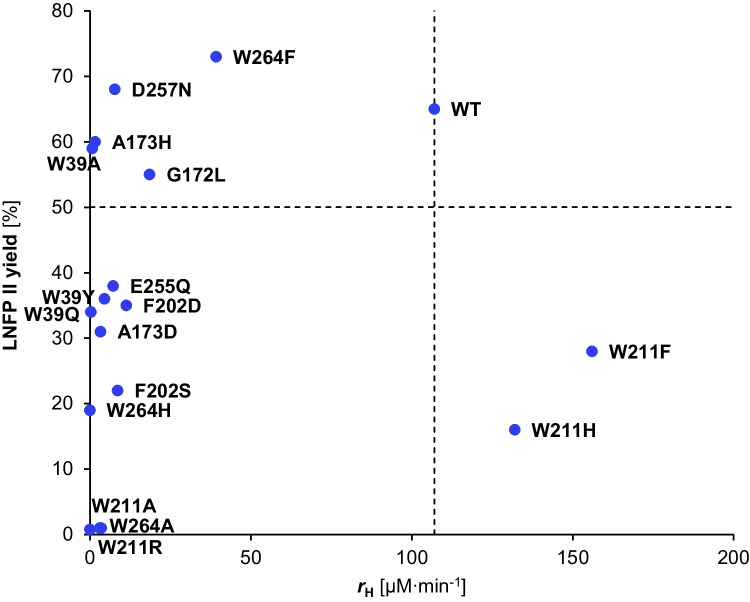
Overview of *Sp*GH29^C^ mutant performance displayed as maximum transglycosylation yields (% LNFP II) vs. hydrolase activity (*r*_H_). In this case, where the WT transglycosylation yield is high, highly transglycosylating mutants are defined as those yielding over 50% LNFP II and display a rate of hydrolysis (*r*_H_) below that of the WT

As guided by the *BindScan* simulations, the results clearly indicate that the hydrolytic activity of the enzyme variants was compromised (both primary and secondary hydrolysis, as these are not distinguishable with the analytical methods employed here) but transglycosylation could in many cases be recovered (Fig. 7). Below, we discuss the molecular basis of the mutations, starting with mutants predicted to decrease hydrolysis via alteration of the active site electrostatic potential, before gradually moving into mutants where altered acceptor binding played a role.

The highest transglycosylation yield was obtained with W264F, despite the fact that its hydrolysis rate was higher than what was observed for many other variants (Table 2, Fig. 7). Most likely, an important reduction in secondary hydrolysis took place, while maintaining a transglycosylation rate (*r*_T_) almost at the level of the WT, which had the highest rates for both reactions (Table 2). W264 is located at the − 1 subsite, establishing CH-π interactions with the donor fucose (Fig. 2f–g). Disrupting enzyme–substrate interactions at negative subsites in retaining GHs is a rational strategy to favor transglycosylation via destabilization of the Michaelis complex of the secondary hydrolysis (Bissaro et al. 2015b). Here, this effect is complemented with a moderate destabilization of the hydrolytic TS as predicted by *BindScan* to achieve the maximum transglycosylating *Sp*GH29^C^ variant. In this way, abolishment or delay of secondary hydrolysis affords a reaction system that does not require tight reaction time control to maintain maximum transglycosylation yields.

W39Q and W39A were the variants with strongest reduction of hydrolytic activity (380-fold and 150-fold, respectively). This is in part due to the direct enzyme substrate interactions established at the active site (Fig. 2f–g) and to the reduction of the electrostatic potential gradient at the scissile glycosidic bond (Fig. 3b). Transglycosylation was recovered only for W39A (59% LNFP II yield) indicating that maintaining hydrophobicity at the acceptor binding pocket is important (W39Y also improved the transglycosylation rate compared to W39A). Also, the E255Q mutant showed a significant reduction of hydrolytic activity, as predicted, but in this case, transglycosylation could not be recovered (Table 2) because it is a second-shell amino acid that does not participate in direct enzyme–substrate interactions (indeed, it was not identified by *BindScan* as a spot for acceptor binding; Fig. 3 and Table 1).

In certain cases, a spot was predicted by *BindScan* to decrease hydrolysis (Fig. 3b), but also to decrease the desired acceptor binding (Fig. 3c–d). For instance, the hydrolytic rate of variant D257N was significantly reduced from the wild type, as predicted by *BindScan* (Fig. 3b), whereas the transglycosylation rate was only halved (Table 2), achieving an ascending maximum of 68% LNFP II formation yield after 24 h with very low secondary hydrolysis (Fig. 4c–d). For this variant, the predicted decrease in binding affinity of the LNB was not detrimental to recovering transglycosylation efficiency.

For some of the mutants, targeting positions A173 and F202 (Fig. 3c–d), the high LNFP II formation levels were a result of a predicted improvement in acceptor binding only. This is the case for variant A173H that displayed the highest T/H among the well-performing variants: while its *r*_T_ was less than threefold lower than that of the WT, its *r*_H_ was reduced almost 70-fold (Table 2). This resulted in a constant product build-up over the first 5 h of reaction (56% LNFP II yield), followed by a slight increase to 24 h (60% LNFP II yield). The corresponding release of Fuc was less than 2% (Fig. 5c–d). Computational analyses revealed that the His residue forms a new hydrogen bond with the endocyclic oxygen of the GlcNAc residue located at the proximal + 1* subsite, thus keeping the acceptor in place for transglycosylation (Fig. 3e). F202 was also identified by *BindScan* as leading to increased acceptor binding (Fig. 3c–d, Table 1). However, the variants F202D and F202S did not display any transglycosylation improvement over the WT enzyme (same T/H ratio, Table 2), indicating that an additional H-bond with the acceptor at a distant + 2’ subsite (Fig. S6a) was not enough to favor transglycosylation over hydrolysis.

For all the completely inactive variants, more subtle amino acid substitutions resulted in active enzymes, and even some with better transglycosylation yield performance as outlined above, indicating an important role of certain amino acid properties to maintain enzyme function. For the two Trp residues W211 and W264, substitution to Phe was more beneficial for transglycosylation than substitution to His, indicating a prevalent role of hydrophobic interactions with the acceptor (W211) and the donor (W264) at these two positions to maintain enzymatic activity (Fig. 2f–g). It is commonly not trivial to predict whether a Trp-to-Phe or a Trp-to-His substitution will be more beneficial for transglycosylation (Vuillemin et al. 2021; Moya-Gonzálvez et al. 2024). In contrast, comparably drastic changes in position W39 were less detrimental to activity (Table 2).

We have previously observed that yields were consistently lower in the enzymatic synthesis of LNFP III as compared to that of LNFP II (Zeuner et al. 2018), suggesting that LNT is a better acceptor molecule for these GH29B enzymes compared to LNnT. In this scenario, some of the designed variants may improve the recognition of this acceptor. Indeed, while transglycosylation yields for LNFP III were lower than for LNFP II, most of the tested variants performed better than the WT enzyme, and neither displayed a transient product maximum like the WT. For F202D, where yields remained inferior to the WT within the reaction time, the *BindScan* simulations indicated that polar amino acid substitutions at F202 increased the stability of the LacNAc acceptor in the binding pocket via the formation of a H-bond with the exocyclic CH_2_OH group of Gal ring in subsite + 2’. This may contribute to keeping the GlcNAc ring tightly bond at the + 1* subsite for the fucosyl transfer reaction (Fig. S6b), despite as mentioned for the reactions towards LNFP II that this H-bond is not enough to recover high transglycosylation rates.

We also performed additional *BindScan* simulations with different acceptors aimed at designing enzyme variants with controlled regioselectivity. Our results indicate that mutations at position W211 gave rise to altered regioselectivity in the reaction towards LNFP II. In the WT enzyme, W211 establishes CH-π interactions with the Gal ring located at the + 2’ subsite in the Lewis^A^ complex (Hobbs et al. 2019) and in the modelled structure of the transglycosylation reaction intermediate (Fig. 3). This places the GlcNAc ring at subsite + 1* ready for the fucosyl transfer reaction. *BindScan* predicted this position as cold spot (Fig. 3c-–d; Table 1) suggesting that mutations at W211 may destabilize this acceptor substrate orientation. *BindScan* simulations reveal that upon mutation, W211 can relieve space to accommodate the Gal ring at subsite + 1 properly oriented for the fucosyl transfer reaction (Fig. 3f). Indeed, W211 is predicted to be a hot spot that stabilizes the acceptor in this new orientation in a separate *BindScan* simulation with LacNAc acceptor (Fig. S7 and Fig. S8). Unlike W39 and W264, W211 is not conserved in GH29A, where regioselectivity in transglycosylation is generally lower (Saumonneau et al. 2016; Zeuner and Meyer 2020; Yang et al. 2024a). In the synthesis of LNFP III, the A173H variant gave the highest yield and T/H, but also produced the highest percentage of linear isomers (13%). Indeed, *BindScan* predicts an important stabilization of the linear isomer for mutants at this position (Fig. S7 and Fig. S8).

Using the *BindScan* protocol, we achieved enzyme variants with improved transglycosylation at 1:1 donor–acceptor ratio, not only slightly surpassing the maximum product yield of the WT enzyme, but more importantly strongly reducing secondary hydrolysis, which allows product accumulation at longer reaction times now becoming practical for preparative enzymatic synthesis of the target compounds. Based on our results, a transglycosylation regime window could be identified for variants with strongly reduced, yet not completely abolished, hydrolytic activity (Fig. 7). This is similar to previous transglycosidase engineering campaigns (Castejón-Vilatersana et al. 2021), but with the particularity that the WT *Sp*GH29^C^ enzyme already exhibits pronounced transglycosylation activity.

Our findings indicate that GH-catalyzed transglycosylation can be optimized guided by computational design as a compromise between glycosidic bond cleavage reduction to delay secondary hydrolysis and proper acceptor binding to favor transglycosylation to render high product yields. Engineering regioselectivity in transglycosylation is the next challenge. Here, we show an initial attempt at rational design of regioselectivity with *BindScan*. Differential binding affinity measurements in silico for different acceptor orientations can guide the selection of regioselective spots in transglycosidase design. In this way, we successfully identified W211 position as a specific spot in *Sp*GH29^C^ that is important in controlling formation of branched vs. linear LNFP regioisomers. Further, F202 and D257 variants appeared to improve regioselectivity in the formation of LNFP III.

## Supplementary information

Below is the link to the electronic supplementary material.

ESM 1 (PDF 3.27 MB)

## Data Availability

All data supporting the findings of this study are available within the paper and its Supplementary Information.
