# Supplementary material for: Computationally guided enzyme engineering for regioselective synthesis of fucosylated human milk oligosaccharides
Source: Appl Microbiol Biotechnol. 2026 May 14;110:205. doi: 10.1007/s00253-026-13860-8 (PMC13342150; doi:10.1007/s00253-026-13860-8)
Supplement: Supplementary file 1 [file 253_2026_13860_MOESM1_ESM.pdf]

Supplementary Information

for

Computationally guided enzyme engineering for regioselective synthesis of fucosylated human milk oligosaccharides

Yaya Yang[1], Aitor Vega[1,2], Jesper Holck[1], Antoni Planas[2,3], Xevi Biarnés[2]*, Birgitte Zeuner[1]*

[1]Section for Protein Chemistry and Enzyme Technology, Department of Biotechnology and Biomedicine, Technical University of Denmark, Kgs. Lyngby, Denmark.

[2]Laboratory of Biochemistry, Institut Químic de Sarrià, University Ramon Llull, Barcelona, Spain.

[3]Chemistry section, Royal Academy of Sciences and Arts of Barcelona, Barcelona, Spain.

*Corresponding authors; e-mails: xevi.biarnes@iqs.url.edu, bzeu@dtu.dk

Table S1. Forward (_f) and reverse (_r) primers for site-directed mutagenesis of *Sp*GH29^{C}. Mutated codons are underlined.

Mutant	Primer sequence (5' to 3')
**A173H_f**	GTG TGG ATG GAT GGT CAT CGT GGT GAA GGT GCG C
**A173D_r**	GTG TGG ATG GAT GGT GAT CGT GGT GAA GGT GCG C
**A173_r**	ACC ATC CAT CCA CAC TTC CGC GAA TTT GCC CG
**D257N_f**	AGC ATT GGT GAA GCG AAC GTG AGC ATC CGT CCG
**D257R_f**	AGC ATT GGT GAA GCG CGT GTG AGC ATC CGT CCG
**D257_r**	CGC TTC ACC AAT GCT GAA GAT GGT GCC GCT C
**W264F_f**	AGC ATC CGT CCG GGC TTC TTT TAT CAC GAG GAC CAG GAT C
**W264H_f**	AGC ATC CGT CCG GGC CAT TTT TAT CAC GAG GAC CAG GAT C
**W264A_f**	AGC ATC CGT CCG GGC GCG TTT TAT CAC GAG GAC CAG GAT C
**W264R_f**	AGC ATC CGT CCG GGC CGT TTT TAT CAC GAG GAC CAG GAT C
**W264_r**	GCC CGG ACG GAT GCT CAC ATC CGC TTC ACC AAT GC
**F202D_f**	GGC GAT TGC CTG ATT GAT AGC ACC GAG GGT ACC A
**F202S_f**	GGC GAT TGC CTG ATT AGC AGC ACC GAG GGT ACC A
**F202_r**	AAT CAG GCA ATC GCC TTG CAG GTC ACG GAT GG
**W39A_f**	TTC TAC GAC CAA GAA GCG GGT ACC GGC CAG GA
**W39Q_f**	TTC TAC GAC CAA GAA CAG GGT ACC GGC CAG GA
**W39Y_f**	TTC TAC GAC CAA GAA TAT GGT ACC GGC CAG GA
**W39_r**	TTC TTG GTC GTA GAA GGT GTT CGG GCC AAA
**W211A_f**	GGT ACC AGC ATC CGT GCG ATT GGC AAC GAA CGT GGT T
**W211H_f**	GGT ACC AGC ATC CGT CAT ATT GGC AAC GAA CGT GGT T
**W211F_f**	GGT ACC AGC ATC CGT TTT ATT GGC AAC GAA CGT GGT T
**W211R_f**	GGT ACC AGC ATC CGT CGT ATT GGC AAC GAA CGT GGT T
**W211_r**	ACG GAT GCT GGT ACC CTC GGT GCT AAA AAT CAG G
**E255K_f**	ATC TTC AGC ATT GGT AAA GCG GAT GTG AGC ATC
**E255Q_f**	ATC TTC AGC ATT GGT CAG GCG GAT GTG AGC ATC
**E255_r**	ACC AAT GCT GAA GAT GGT GCC GCT CGG G
**G172L_f**	GAA GTG TGG ATG GAT CTG GCG CGT GGT GAA GGT
**G172W_f**	GAA GTG TGG ATG GAT TGG GCG CGT GGT GAA GGT
**G172_r**	ATC CAT CCA CAC TTC CGC GAA TTT GCC CGC
**Inactivation of acid/base residue**	
**E215A_f**	CGT TGG ATT GGC AAC GCG CGT GGT TAT GCG GGC G
**E215Q_f**	CGT TGG ATT GGC AAC CAG CGT GGT TAT GCG GGC G
**E215_r**	GTT GCC AAT CCA ACG GAT GCT GGT ACC CTC G

Table S2. Expression yields for all variants of *Sp*GH29^{C} given as mg purified protein per liter of fermentation.

**Variant**	Expression yield (mg/L)
WT	35
W39A	50
W39Q	35
W39Y	55
G172L	54
G172W	64
A173D	44
A173H	23
F202D	66
F202S	65
W211A	76
W211F	99
W211H	79
W211R	118
E215A	15
E215Q	26
E255K	96
E255Q	72
D257N	35
D257R	100
W264A	89
W264F	24
W264H	27
W264R	100

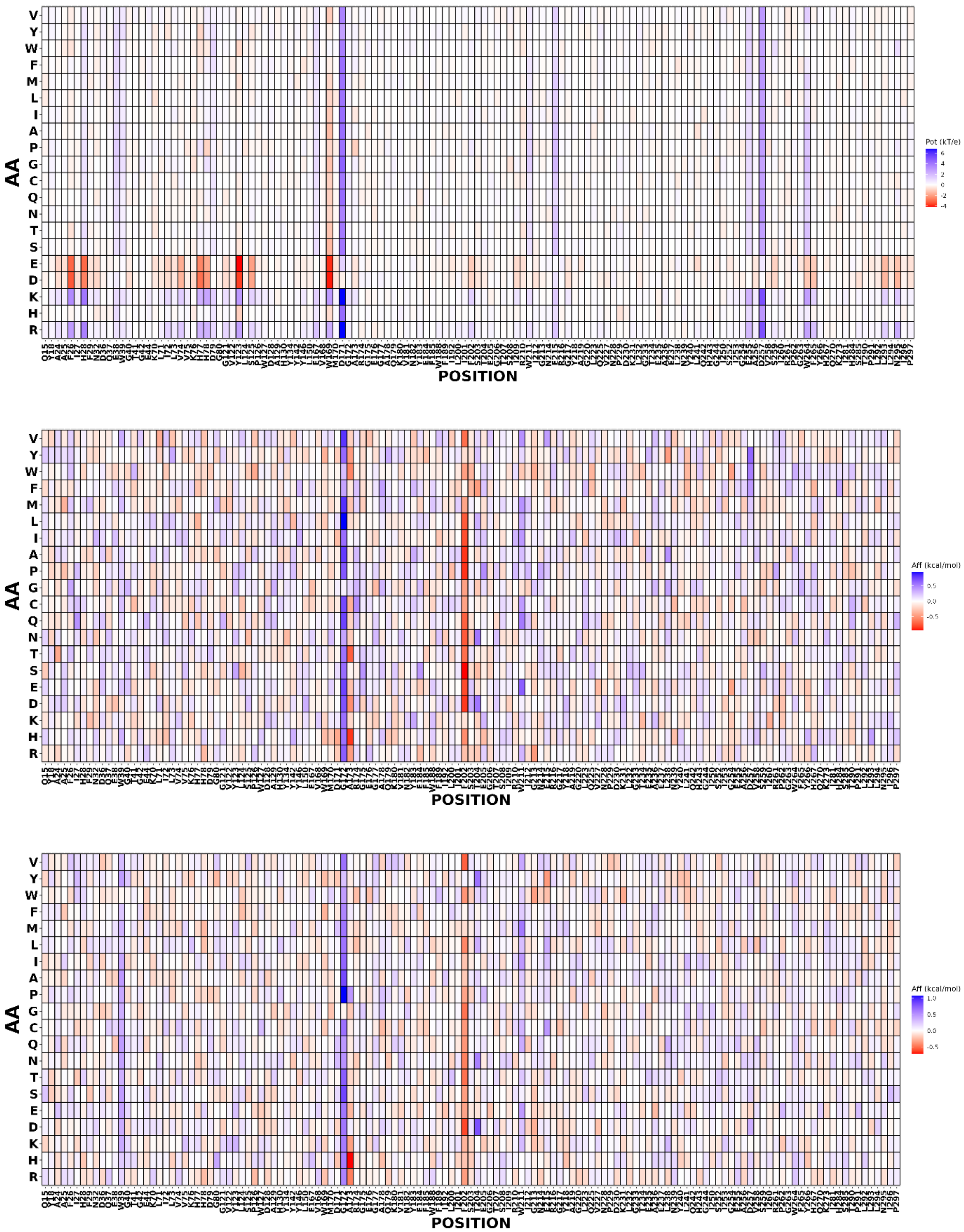

Fig. S1. *BindScan* results on the effect of *Sp*GH29^{C} point mutations on the electrostatic potential gradient at the active site (top), binding affinity of the LNB acceptor in the orientation leading to the LNFP II isomer(middle) and binding affinity of LacNAc acceptor in the orientation leading to the LNFP III isomer (bottom). In the heatmap, for each sequence position (horizontal axis) the effect of point mutations to each of the 19 natural amino acids is shown in a color scale ranging from red (gain of function) to blue (loss of function). Reference values for the wild type are shown in white.

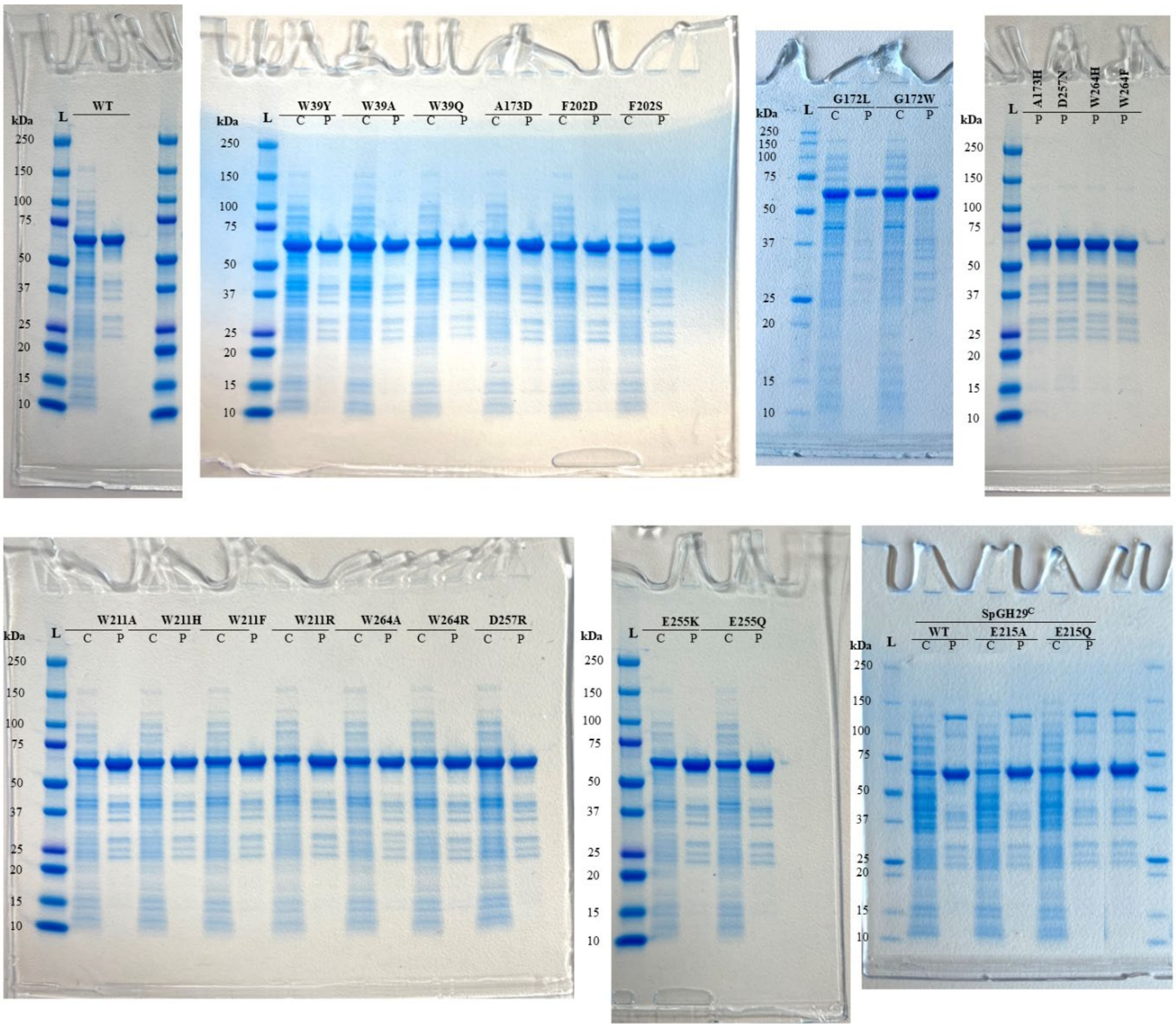

Fig. S2. SDS-PAGE of the purified WT *Sp*GH29^{C} and its variants. Lane L: ladder; lane C: crude enzyme; lane P: purified enzyme after purification and desalting. The WT in the very last SDS-PAGE (marked “(WT)”) was not used in this work; this SDS-PAGE contains the acid/base inactive mutants.

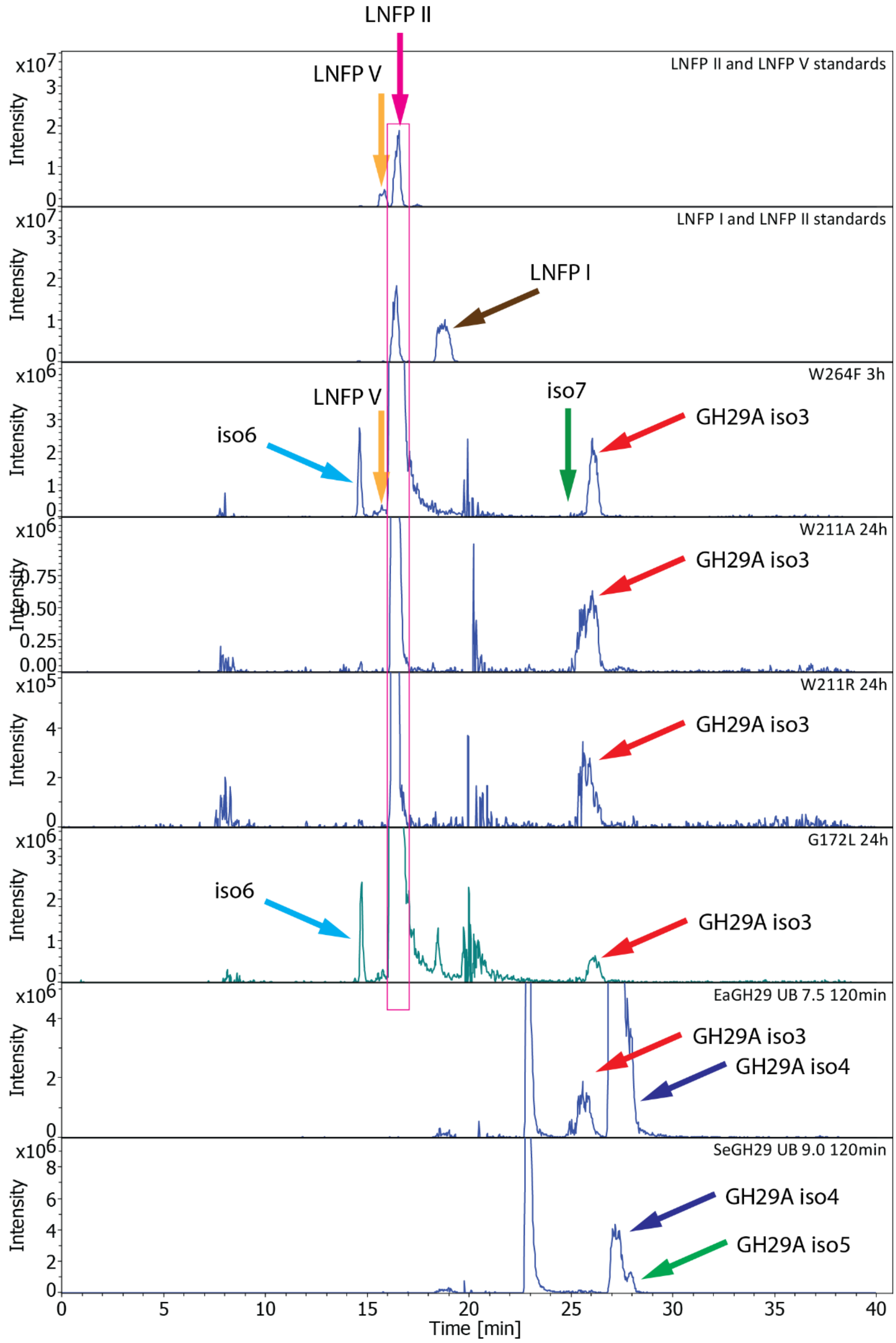

Fig. S3. LC-ESI-MS extracted ion chromatograms of 852 *m/z* corresponding to the deprotonated adduct of external standards of LNFP I, LNFP II, and LNFP V (two top panels), and of selected samples from the reactions of 3FL and LNT catalyzed by *Sp*GH29^{C} W264F, W211A, W211R, and G172L. The two lower panels display samples of the reaction between 2'fucosyllactose (2'FL) and LNT catalyzed by GH29A fucosidases EaGH29 and SeGH29 resulting in linear LNFP I isomers, i.e. unbranched, as published previously [1], of which the isomer named iso3 was also detected in the current work, while iso4 and iso5 formed by the GH29A enzymes were not. The unidentified isomer iso6 present in samples of W264F and G172L may well be a branched isomer, judging from the retention time being close to LNFP II, LNFP III (Fig. S3), and LNFP V, which are all branched.

Table S3. Percentages of LNFP II and its regioisomers LNFP V, iso6, iso7, and iso3 quantified by LC-ESI-MS in the reaction samples with the highest transglycosylation yields for each enzyme variant. The column "total isomers" sums up the percentage of regioisomers and numbers highlighted in bold correspond to samples with >5% of the regioisomers and are thus indicated in the main text table (Table 2).

**Sample**	**LNFP II**	**LNFP V**	**iso6**	**iso7**	**iso3**	**Total isomers**
*Sp*GH29C_WT	98.0%	0.5%	1.1%	0.1%	0.4%	2.0%
*Sp*GH29C_W39A	98.2%	0.6%	1.1%	0.0%	0.1%	1.8%
*Sp*GH29C_W39Q	98.0%	0.6%	1.2%	0.0%	0.1%	2.0%
*Sp*GH29C_W39Y	97.4%	0.9%	1.3%	0.3%	0.2%	2.6%
*Sp*GH29C_G172L	98.6%	0.0%	0.7%	0.0%	0.7%	1.4%
*Sp*GH29C_A173H	96.8%	0.7%	1.3%	0.0%	1.1%	3.2%
*Sp*GH29C_A173D	99.0%	0.1%	0.6%	0.0%	0.3%	1.0%
*Sp*GH29C_F202D	97.5%	0.4%	1.0%	0.0%	1.1%	2.5%
*Sp*GH29C_F202S	97.8%	0.3%	0.7%	0.1%	1.2%	2.2%
*Sp*GH29C_W211A	84.9%	0.0%	0.3%	0.3%	14.4%	**15.1%**
*Sp*GH29C_W211H	93.9%	0.2%	0.5%	0.1%	5.2%	**6.1%**
*Sp*GH29C_W211F	94.8%	0.5%	0.9%	0.1%	3.7%	**5.2%**
*Sp*GH29C_W211R	87.1%	0.0%	0.3%	0.3%	12.4%	**12.9%**
*Sp*GH29C_E255Q	98.2%	0.5%	1.1%	0.0%	0.2%	1.8%
*Sp*GH29C_D257N	96.5%	1.7%	1.2%	0.1%	0.6%	3.5%
*Sp*GH29C_W264F	94.8%	0.4%	1.5%	0.1%	3.3%	**5.2%**
*Sp*GH29C_W264H	99.1%	0.2%	0.6%	0.0%	0.1%	0.9%
*Sp*GH29C_W264A	99.2%	0.0%	0.5%	0.1%	0.2%	0.8%

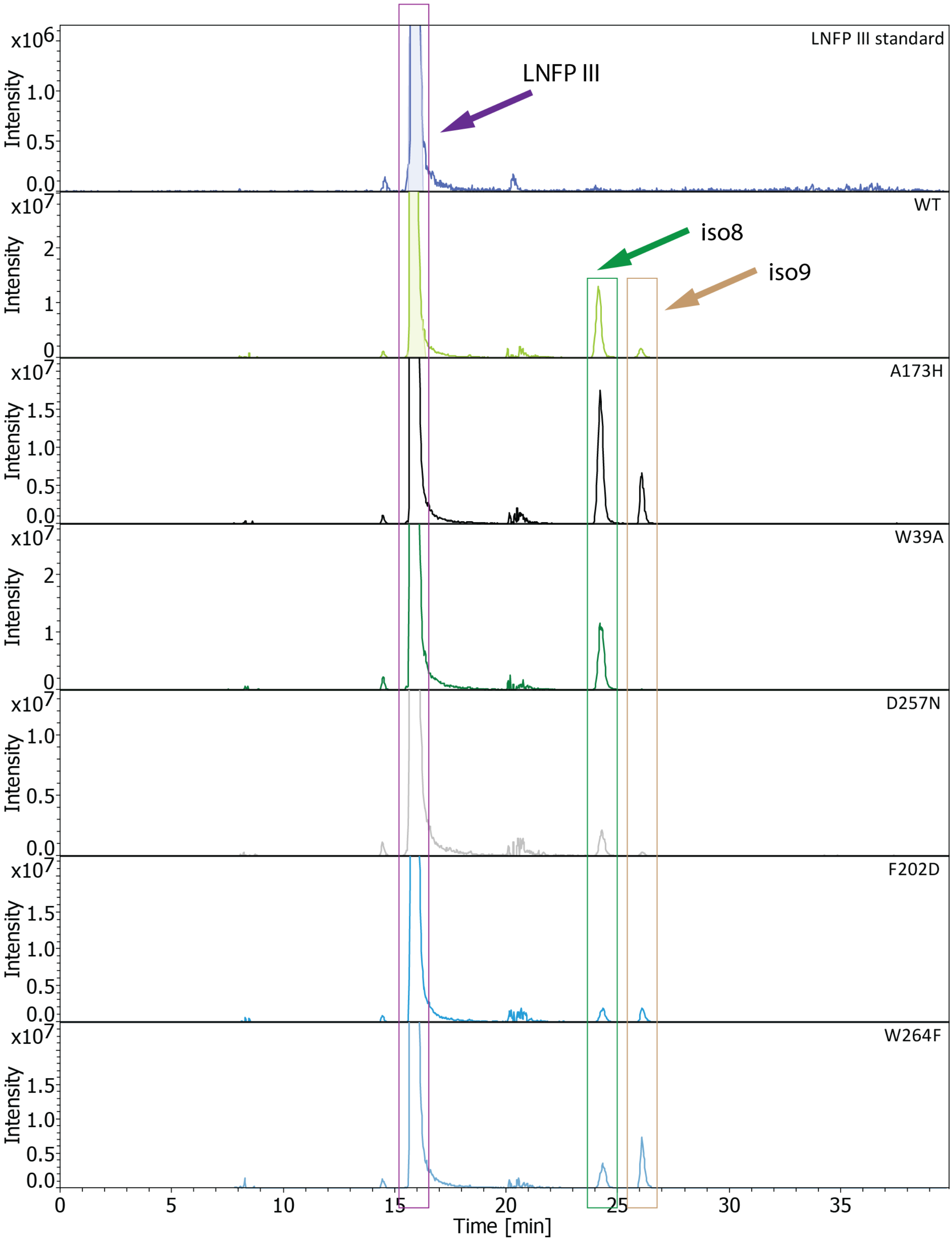

Fig. S4. LC-ESI-MS extracted ion chromatograms of 852 *m/z* corresponding to the deprotonated adduct of the external standards of LNFP III (top panel), and of selected samples from the reactions of 3FL and LNnT catalyzed by *Sp*GH29^{C} WT, A174H, W39A, D257N, F202D, and W264F. In addition to LNFP III, two isomers (iso8 and iso9) were detected. Isomers iso8 and iso9 both fragment in MS^2 as linear, i.e. unbranched, isomers with the diagnostic fragment 325 *m/z* (Fig. S4).

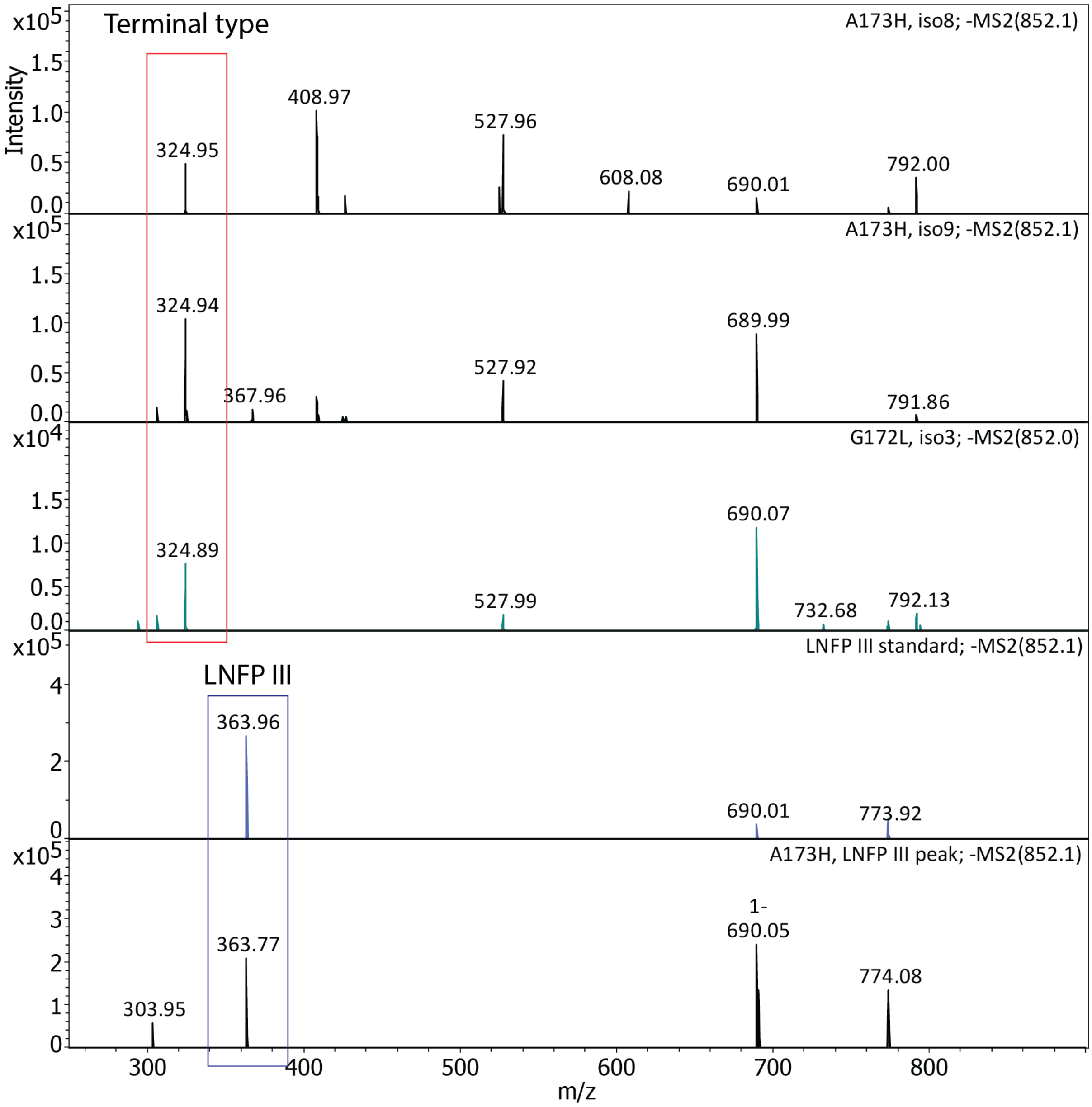

Fig. S5. MS^2 fragmentation of selected 852 *m/z* peaks in Fig. S3, and of the G172L iso3 peak in Fig. S2. Fragmentation of other relevant peaks in Fig. S2 was presented previously [1,2]. The diagnostic fragments [3] of LNFP III (364 *m/z*) and of a linear LNFP isomer resembling LNFP I (325 *m/z*) have been highlighted in boxes.

Table S4. Percentages of LNFP III and its linear regioisomers iso8 and iso9 quantified by LC-ESI-MS in the reaction samples with the highest transglycosylation yields for each enzyme variant. The column "total isomers" sums up the percentage of regioisomers and numbers highlighted in bold correspond to samples with >5% of the regioisomers and are thus indicated in the main text table (Table 3).

Sample	LNFP III	iso8	iso9	Total isomers
SpGH29C_WT	92.2%	6.8%	1.0%	**7.8%**
SpGH29C_A173H	86.9%	10.0%	3.1%	**13.1%**
SpGH29C_W39A	93.5%	6.3%	0.1%	**6.5%**
SpGH29C_D257N	98.5%	1.2%	0.2%	1.5%
SpGH29C_F202D	97.6%	1.3%	1.1%	2.4%
SpGH29C_W264F	94.6%	2.2%	3.2%	**5.4%**

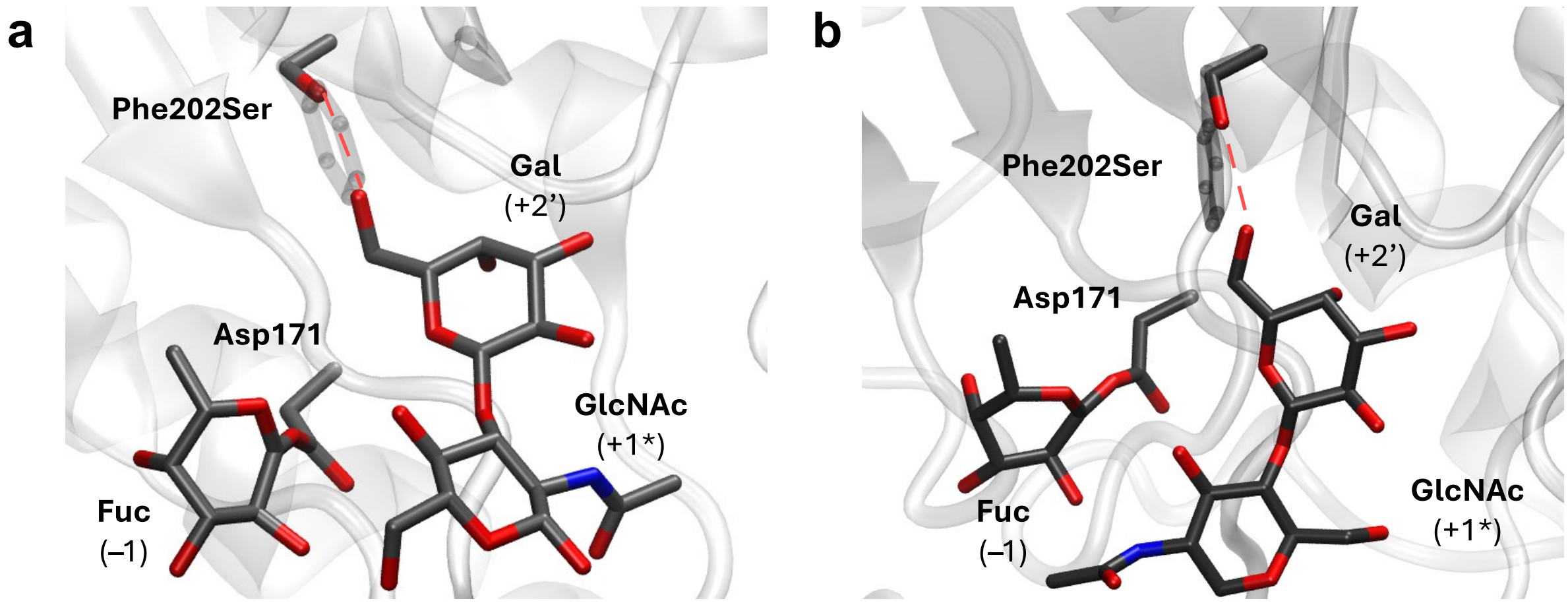

Fig. S6. Magnified representation of the catalytic site of *Sp*GH29^{C} F202S mutant in complex with a) LNB in the LNFP II isomer formation orientation and b) LacNAc in the LNFP III isomer formation orientation.

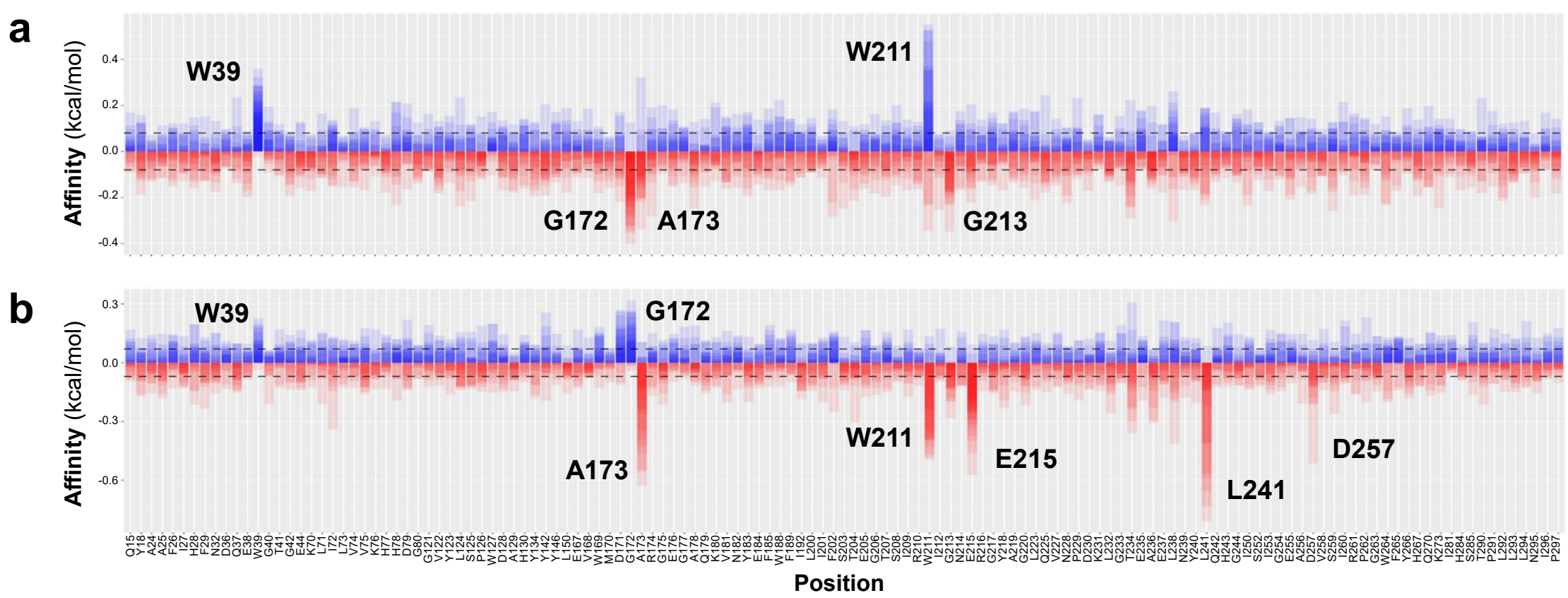

Fig. S7. *BindScan* prediction of mutation spots in *Sp*GH29^{C} that modulate a) binding of the LNB acceptor in the orientation leading to the linear isomer and b) binding of the LacNAc acceptor in the orientation leading to the linear isomer.

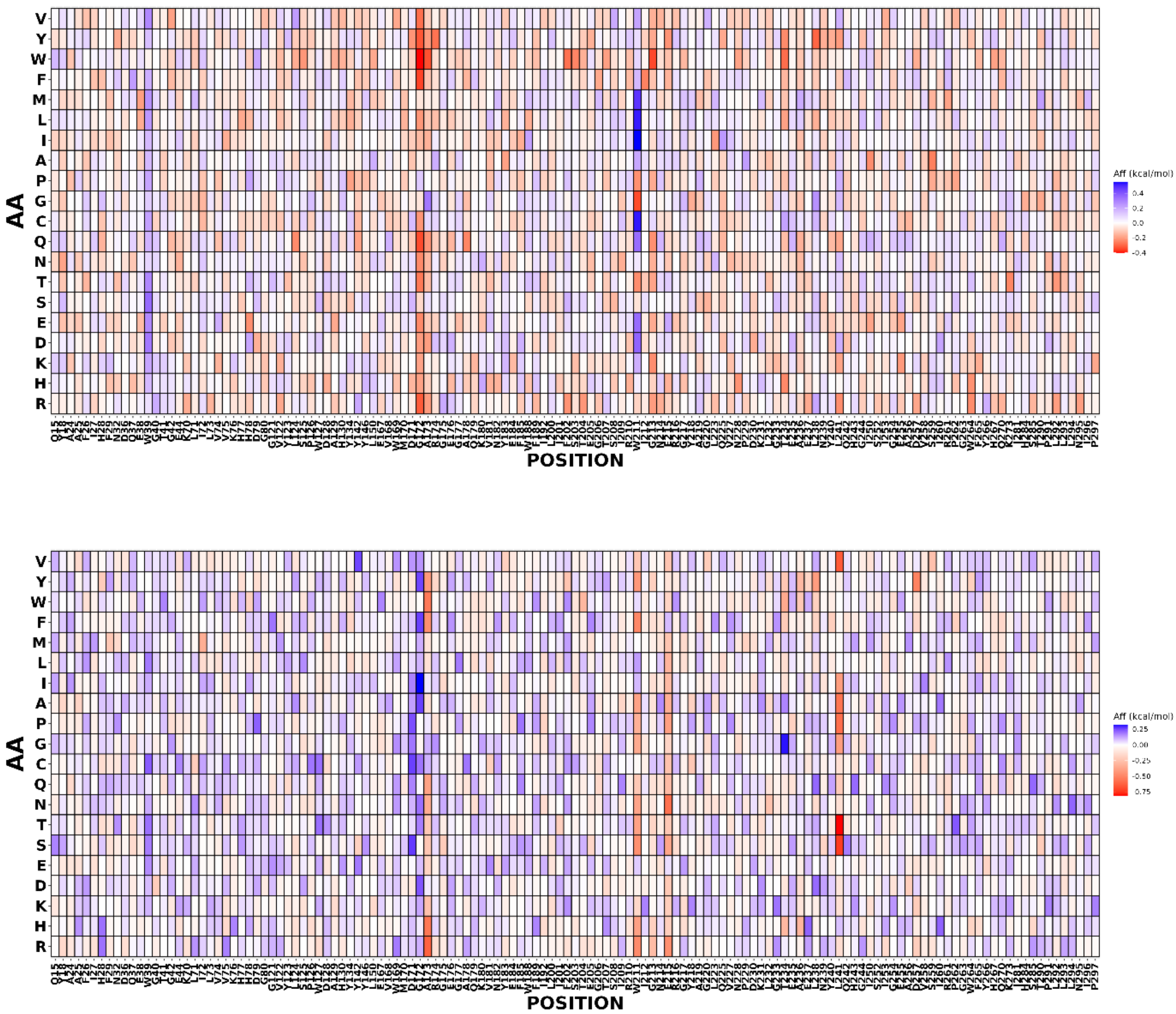

Fig. S8. *BindScan* results on the effect of *Sp*GH29^{C} point mutations on the binding affinity of the LNB acceptor in the orientation leading to the linear isomer (top) and the binding affinity of the LacNAc acceptor in the orientation leading to the linear isomer (bottom). In the heatmap, for each sequence position (horizontal axis) the effect of point mutations to each of the 19 natural amino acids is shown in a color scale ranging from red (increased affinity) to blue (decreased affinity). Reference values for the wild type are shown in white.

References for Supplementary Information

[1] Yang Y, Holck J, Thorhallsson AT, Hunt CJ, Yang H, Morth JP, et al. Structural elucidation and characterization of GH29A α-l-fucosidases and the effect of pH on their transglycosylation. FEBS Journal 2024. https://doi.org/10.1111/febs.17347.

[2] Yang Y, Thorhallsson AT, Rovira C, Holck J, Meyer AS, Yang H, et al. Improved Enzymatic Production of the Fucosylated Human Milk Oligosaccharide LNFP II with GH29B α-1,3/4-l-Fucosidases. J Agric Food Chem 2024;72:11013–28. https://doi.org/10.1021/acs.jafc.4c01547.

[3] Pfenninger A, Karas M, Finke B, Stahl B. Structural analysis of underivatized neutral human milk oligosaccharides in the negative ion mode by nano-electrospray MSn (Part 2: Application to isomeric mixtures). J Am Soc Mass Spectrom 2002;13:1341–8. https://doi.org/10.1016/s1044-0305(02)00646-3.
